# TOR Is a Negative Regulator of Autophagy in *Arabidopsis thaliana*

**DOI:** 10.1371/journal.pone.0011883

**Published:** 2010-07-29

**Authors:** Yimo Liu, Diane C. Bassham

**Affiliations:** 1 Interdepartmental Genetics Program, Iowa State University, Ames, Iowa, United States of America; 2 Department of Genetics, Development and Cell Biology, Iowa State University, Ames, Iowa, United States of America; Plant Sciences Institute, Iowa State University, Ames, Iowa, United States of America; University of Heidelberg, Germany

## Abstract

**Background:**

Autophagy is a protein degradation process by which cells recycle cytoplasmic contents under stress conditions or during senescence; a basal level of housekeeping autophagy also occurs under non-stressed conditions. Although a number of genes that function in autophagy (*ATG* genes) have been identified in plants, the upstream components that regulate the plant autophagy pathway are still obscure. Target of rapamycin (TOR) is a negative regulator of autophagy in both yeast and animals, and homologs of TOR in plants control plant growth and protein synthesis. However, a role for TOR in regulation of autophagy in plants has not been demonstrated previously.

**Methodology/Principal Findings:**

In this paper we used RNA interference (RNAi) to generate transgenic plants with reduced *AtTOR* transcript level. By observing monodansylcadaverine- (MDC) and GFP-AtATG8e-labeled autophagosomes, these plants were demonstrated to have constitutive *AtATG18a*-dependent autophagy. Reverse transcriptase-PCR also showed increased expression of some *AtATG* genes in the RNAi-*AtTOR* plants. Unlike autophagy induced by starvation or salt stress, an NADPH oxidase inhibitor did not inhibit the constitutive autophagy in the RNAi-*AtTOR* lines, indicating that *AtTOR* is either downstream of or in a parallel pathway to NADPH oxidase.

**Conclusions/Significance:**

Together, our results provide evidence that TOR is a negative regulator of autophagy in plants.

## Introduction

Upon exposure to stress conditions or during senescence, plants degrade cytoplasmic macromolecules inside the vacuole [1] by a process known as macroautophagy, or autophagy for short [2]. Autophagy is highly induced under several types of stress conditions [3], [4], [5], [6], [7], [8], [9]; a basal level of autophagy also exists in cells to constitutively remove unwanted materials [10], [11]. Upon activation of autophagy, a double membrane structure called an autophagosome forms around the cargo to be degraded, and then delivers it to the vacuole where it is broken down and recycled [12]. Several specific functions of autophagy have been characterized in plants, including degradation of aggregated proteins in nutrient-starved plant cells [13] and proteins damaged during oxidative stress [6], [14], and regulation of cell death during pathogen immune responses [15], [16], [17].

In *Arabidopsis thaliana*, many *AuTophaGy (ATG)* genes have now been characterized and found to be essential for autophagy. These have primarily been identified based on sequence similarity to yeast autophagy genes. For example, in yeast the autophagy proteins Atg2, Atg9 and Atg18 form a functional module, as Atg2 and Atg18 are required for Atg9 cycling [18]. In *Arabidopsis*, an Atg18 homolog, AtATG18a, has been shown to be required for autophagosome formation during multiple environmental stress conditions and also leaf senescence [5]. *AtATG18a* is expressed throughout the plant, with an increase in transcript level observed during conditions that upregulate the autophagy pathway [5], [6]. In RNAi-*AtATG18a* transgenic plants with reduced *AtATG18a* transcript level, autophagosome formation was disrupted and the plants were more sensitive to autophagy-inducing stresses and displayed accelerated senescence [5], [6], [19]. Likewise, Arabidopsis *ATG2* and *ATG9* genes also function in autophagy, suggesting that the role of these genes may be conserved between species [5], [8], [10].

Although the identification and characterization of *ATG* genes in plants has greatly enhanced our understanding of autophagy, the upstream regulatory components in the plant autophagy pathway are still obscure. In Arabidopsis, autophagy is induced by multiple abiotic and biotic stresses, including nutrient deficiency, oxidative, salt and drought stresses and pathogen invasion [5], [6], [16], [17], [19]. A potential role for NADPH oxidase in regulation of autophagy under some conditions has been suggested by inhibitor studies; NADPH oxidase inhibitors block autophagy activation during nutrient and salt stress but not during osmotic stress, indicating that NADPH oxidase-dependent and -independent regulatory pathways may exist [19]. In yeast and animals, target of rapamycin (*TOR*) kinase was shown to be a negative regulator of autophagy [20], [21]. Tor is a phosphatidylinositol 3-kinase-related kinase that functions as a Ser/Thr protein kinase [22]. It is inactivated by rapamycin, which forms a complex with FKBP12 (FK506 binding protein); this complex binds Tor and inhibits Tor activities [23], thus being a valuable tool in determining Tor function. Tor also controls other processes that regulate growth in response to nutrient status, for example control of translation initiation by activating the ribosomal p70 S6 kinase and inhibiting the eukaryotic translation initiation factor 4E binding protein 1 (eIF-4E BP1) [24], [25], [26], [27].

In yeast, two *TOR* genes have been identified; plants, mammals and other eukaryotes have only one *TOR* gene [21]. In both yeast and mammals, two TOR complexes exist, TORC1 and TORC2, each of which contains distinct TOR binding partners. Only TORC1 is sensitive to rapamycin [28]. Some binding partners have been identified, including Raptor [29], which binds to substrates and presents them to Tor for phosphorylation, and LST8 [30], [31], which stabilizes the TOR complex. The Atg1/Atg13 complex, which is required at an early stage of autophagy initation to induce autophagosome formation, has been identified as a Tor substrate in metazoans. Tor functions by phosphorylating Atg13 in a nutrient-dependent manner, although the relationship between and regulation of Tor, Atg1 and Atg13 is different between yeast and metazoans [32], [33], [34], [35].

The TOR protein is conserved in plants [36] and RAPTOR homologues have also been identified in Arabidopsis [37], [38]. However, disruption of the *TOR* gene is lethal and causes an early block in embryo development [36], impeding the analysis of TOR function in plants. In addition, Arabidopsis is insensitive to rapamycin, and this inhibitor therefore cannot be used to study the TOR pathway in this species [36], [39]. Previous research with transgenic Arabidopsis plants with increased or decreased *TOR* expression level [40], [41] showed that growth of root and shoot was correlated with *TOR* expression level, indicating a role in growth regulation. A recent study in the green alga *Chlamydomonas reinhardtii* showed that autophagy is induced upon rapamycin treatment, suggesting that regulation of autophagy by TOR may extend to photosynthetic species[42]. However, whether TOR regulates autophagy in multicellular plants has not been investigated.

In this study, our goal was to investigate whether TOR, a negative regulator of autophagy in yeast and mammals, also plays a role in plant autophagy induction. We demonstrate that RNAi-*AtTOR* plants with decreased expression of *AtTOR* have constitutive autophagy and increased expression of some *ATG* genes, even in the absence of stress conditions. This constitutive autophagy is dependent on the autophagy gene *AtATG18a*. We also show that, unlike stress-induced autophagy, an NADPH oxidase inhibitor does not inhibit the constitutive autophagy in these plants, indicating that *AtTOR* is most likely either downstream of or in a parallel pathway to NADPH oxidase. Taken together, our results indicate that *AtTOR* is a negative regulator of autophagy in *Arabidopsis thaliana*.

## Results

### Generation of RNAi-*AtTOR* transgenic plants

**Figure 1:**
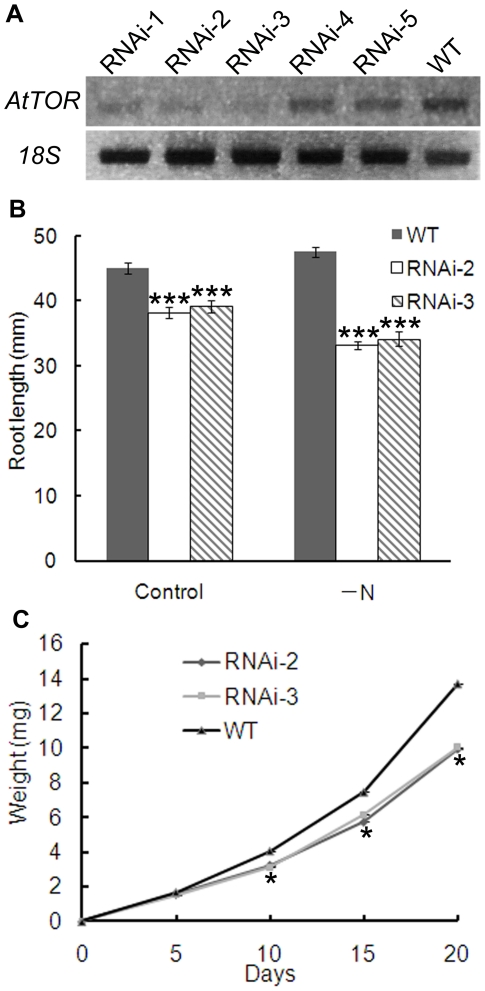
Generation of RNAi-*AtTOR* transgenic lines. **A** Total RNA was extracted from 7-day-old WT and five independent RNAi-*AtTOR* lines, RNAi-1 to RNAi-5, followed by RT-PCR analysis. 18S was used as a loading control. **B** The root length of 10-day-old WT, RNAi-2, RNAi-3 seedlings was measured on both MS medium and MS medium lacking nitrogen (MS-N). **C** The fresh weight of WT, RNAi-2, RNAi-3 seedlings grown on MS medium was measured at the stated times. *** indicates P<0.001; ** indicates P<0.01 and * indicates P<0.05. Error bars indicate standard error.

Because disruption of the *AtTOR* gene (At1g50030) is embryo lethal [36], to study the role of *AtTOR* in autophagy, RNA interference (RNAi) was used to generate plants with reduced *AtTOR* transcript level. An RNAi-*AtTOR* construct was made by linking two inverted *AtTOR* gene specific regions with a 1 kb *GUS* spacer and driven by the Cauliflower Mosaic Virus *35S* promoter [43]. *Arabidopsis thaliana* plants were transformed with this RNAi-*AtTOR* construct using the floral dip method [44] and screened for kanamycin resistance. Transformants with reduced *AtTOR* transcript level were identified by RT-PCR using *AtTOR* gene specific primers (Figure 1A). Out of the 5 independent transgenic lines shown, named RNAi-1 to 5, lines RNAi-2 and RNAi-3 showed the greatest reduction in *AtTOR* transcript compared with the WT control, and were therefore selected for further experiments. All experiments were performed using T2 seedlings, which showed reproducible phenotypes that correlated with residual *AtTOR* expression level. Within a transgenic line, all individuals showed consistent AtTOR expression levels and phenotypes.

Previous research has shown that plants with a partially silenced *AtTOR* gene show reduced growth (e.g. shorter root and shoot length, smaller rosette leaves) [40], [41]. To confirm that the phenotype of the RNAi lines generated were consistent with previous reports, the root length and the fresh weight of seedlings were measured. WT, RNAi-2 and RNAi-3 seeds were germinated and grown on MS medium for 10 days with the plates oriented perpendicular to the ground. The RNAi plants were found to have a small but statistically significant decrease in root length when compared with WT (Figure 1B),which is consistent with previous reports [40]. In yeast, *TOR* regulates growth and protein synthesis in response to nutrient availability [45], [46]; therefore 10-day root length under nitrogen starvation conditions was also analyzed (Figure 1B). The root length of RNAi seedlings was not only decreased compared with WT, but also decreased still further under nitrogen starvation conditions compared with control nutrient-rich conditions.

To measure the fresh weight, RNAi-2, RNAi-3 and WT seedlings grown on MS media were weighed at 5, 10, 15 and 20 days (Figure 1C). From 10 days onward, both RNAi lines had a decreased fresh weight compared with the WT seedlings. To further investigate potential phenotypes of older plants in soil, RNAi-2 and WT plants grown in soil were observed throughout their lifespan. No significant differences were observed between RNAi and WT plants in seed volume, flower time and rosette size (data not shown). This differs from previous reports of reduced growth of *TOR* RNAi plants, possibly due to differences in growth conditions or extent of *TOR* silencing [40].

### RNAi-*AtTOR* plants have constitutive autophagy

**Figure 2:**
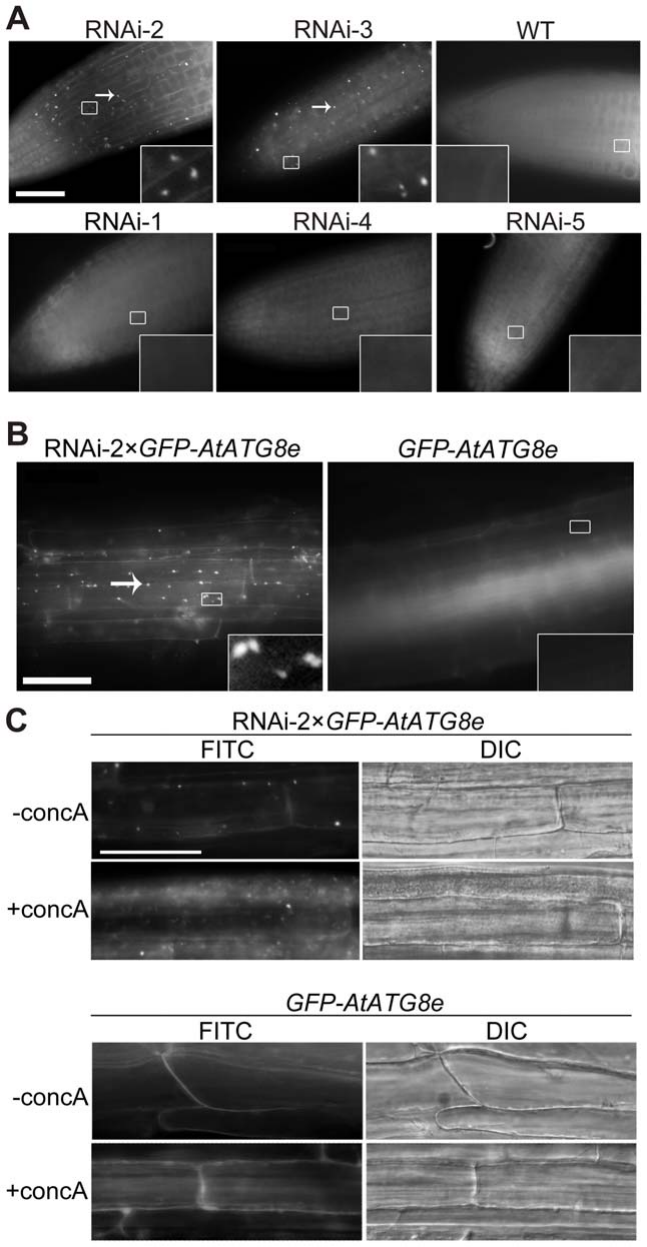
RNAi-*AtTOR* plants have constitutive autophagy. **A** Seven-day-old WT and RNAi-1 to RNAi-5 seedlings were stained with MDC and observed by fluorescence microscopy. Arrows indicate MDC-stained autophagosomes. **B** GFP-*AtATG8e*-labeled autophagosomes were visualized by fluorescence microscopy in 7-day-old GFP-*AtATG8e* and RNAi-2×GFP-*AtATG8e* seedlings. Arrows indicate GFP-labeled autophagosomes. Insets show an enlargement of the boxed areas. Scale bar = 50 µm for main figures, 10 µm for insets. **C** Seven-day-old *GFP-AtATG8e* and RNAi-2× *GFP-AtATG8e* seedlings were transferred to liquid MS medium containing 1 µM concanamycin A (+concA) or DMSO (-concA) as a solvent control for 12 h, followed by both fluorescence and DIC microscopy. Scale bar = 50 µm.

*TOR* is a negative regulator of autophagy in yeast and mammals [20], [21]. Possible effects on autophagy in the absence of stress were therefore investigated in RNAi-*AtTOR* transgenic plants with decreased expression of *AtTOR*. Autophagy can be analyzed in Arabidopsis seedlings by staining with the fluorescent dye monodansylcadaverine (MDC), which selectively labels autophagosomes [47]. WT seeds and transgenic seeds of RNAi lines RNAi-1 to RNAi-5 (Figure 1A) were germinated on MS plates and grown for one week, followed by MDC staining and fluorescence microscopy to visualize autophagy (Figure 2A). In both RNAi-2 and RNAi-3 seedlings, which have the greatest reduction in *AtTOR* transcript level, constitutive autophagy was observed close to the root tip, seen as rapidly moving fluorescent puncta. Very few autophagosomes were seen in transgenic seedlings with less effective reduction of transcript level (RNAi-1, RNAi-4, RNAi-5) and in WT plants. This indicates that the constitutive autophagy phenotype correlates with a greater reduction in *AtTOR* transcript level.

To confirm the constitutive autophagy in RNAi-*AtTOR* plants, transgenic plants expressing the autophagosome marker GFP-AtATG8e [6] were crossed with RNAi-2 plants. Seeds from the cross were germinated on MS plates and grown for one week, then seedlings examined for autophagy activity; the autophagosomes were directly visualized under the fluorescence microscope via GFP fluorescence (Figure 2B). Consistent with the MDC staining result, autophagosomes can be observed even under control conditions in the RNAi-2× *GFP-AtATG8e* plants above the root tip areas, while the *GFP-AtATG8e* transgenic plants have a diffuse GFP signal as expected with very few autophagosomes visible. These results further confirm the constitutive autophagy in RNAi-*AtTOR* plants.

We considered two possibilities to explain the constitutive presence of autophagosomes in RNAi-*AtTOR* plants: either the decreased expression of *AtTOR* leads to the constant formation of autophagosomes, or the RNAi-*AtTOR* plants are deficient in delivery of autophagosomes to the vacuole for degradation. To distinguish between these two possibilities, and to further analyze the subcellular GFP-AtATG8e distribution and fate, the vacuolar H^+^-ATPase inhibitor concanamycin A (conc A) was used to inhibit the degradation of autophagic bodies in the vacuole [9], [48]. Conc A inhibits trafficking of vacuolar proteins and prevents vacuolar protein degradation [9], [49]. Autophagic bodies therefore accumulate in the vacuole in Arabidopsis roots instead of being degraded after treatment with conc A [9]. Seven-day-old *GFP-AtATG8e* and RNAi-2× *GFP-AtATG8e* seedlings were transferred to medium containing 1 µM conc A or dimethyl sulfoxide (DMSO) as a solvent control for 12 h, and then observed by both fluorescence microscopy using a FITC filter and differential interference contrast (DIC) microscopy (Figure 2C). In control conditions, the vacuoles in both RNAi-2× *GFP-AtATG8e* and *GFP-AtATG8e* roots rarely contain any spherical structures in DIC images. *GFP-AtATG8e* roots have a diffuse cytoplasmic GFP signal while RNAi-2× *GFP-AtATG8e* roots contain several punctate GFP-labeled structures in the cytoplasm, consistent with Figure 2B. This indicates that the DSMO solvent did not induce autophagy. After treatment with conc A, the vacuoles in the *GFP-AtATG8e* roots contained some weak and diffuse GFP fluorescence, and as expected, occasionally contained a few GFP puncta corresponding to spherical structures in the DIC images, as plants have a basal housekeeping level of autophagy [10], [11]. In contrast, the vacuoles in the RNAi-2× *GFP-AtATG8e* roots contained many GFP-AtATG8e-labeled puncta and spherical structures in the DIC images which have been shown previously to be autophagic bodies [9]. By comparing the GFP fluorescence with the DIC images in RNAi-2× *GFP-AtATG8e* roots after conc A treatment, most of the GFP-AtATG8e signal and the spherical structures localize inside the vacuole. This suggests that after treatment with conc A, autophagic bodies accumulate in the vacuole in the RNAi-2× *GFP-AtATG8e* roots instead of being degraded. The accumulation of autophagic bodies in the vacuoles of RNAi-2× *GFP-AtATG8e* plants suggests that autophagosomes are formed and successfully transferred into vacuoles in these plants. These results indicate that the constitutive autophagy in RNAi-*AtTOR* seedlings is most likely caused by the increased formation of autophagosomes, rather than a deficiency in delivery to or fusion between autophagosomes and vacuoles. These data therefore suggest that *AtTOR* may negatively regulate autophagy in *Arabidopsis*.

### Some *ATG* genes are up-regulated in the RNAi-*AtTOR* plants

**Figure 3:**
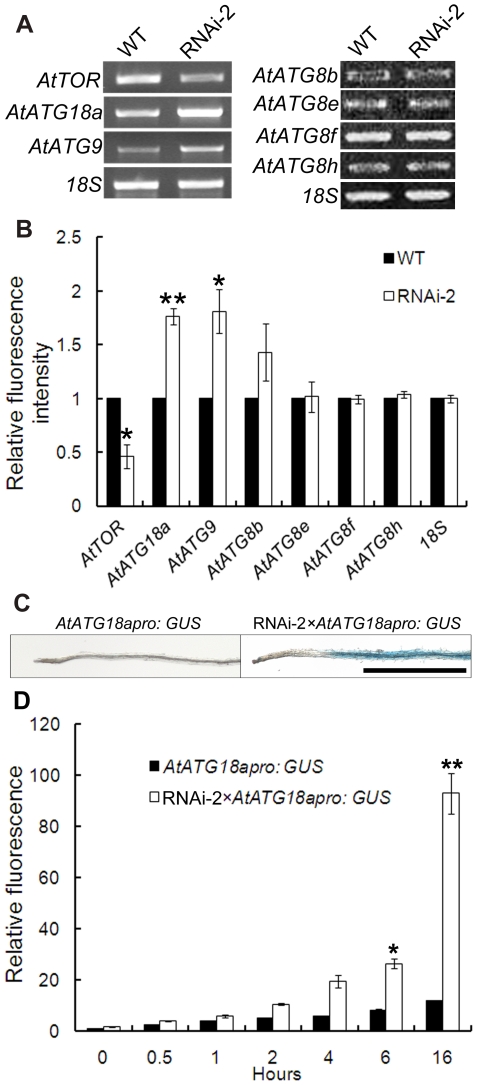
Some *ATG* genes are up-regulated in RNAi-*AtTOR* plants. **A** RNA was extracted from 8 mm of the root tips from seven-day-old WT and RNAi-2 lines, followed by RT-PCR analysis of the indicated genes. 18S was used as a loading control. **B** Densitometry was used to quantify the relative amounts of RT-PCR product from at least three independent replicates, with the wild-type control value set as 1. ** indicates P<0.01 and * indicates P<0.05. Error bars indicate standard error. **C** 7-day-old *AtATG18apro: GUS* and RNAi-2×*AtATG18apro:GUS* seedlings were collected and submerged in GUS staining solution for 16 h, destained in 70% ethanol for 16 h and observed with a light microscope. Scale bar = 1 mm. **D** 7-day-old *AtATG18apro: GUS* and RNAi-2×*AtATG18apro:GUS* root tissue extracts were added to 1 mM MUG solution to assay GUS activity. At 0 h, 0.5 h, 1 h, 2 h, 4 h, 6 h and 16 h, the reactions were terminated with 0.2 M Na_2_CO_3_ followed by measurement of MU fluorescence. Data was collected from 3 independent replicates, with *AtATG18apro: GUS* 0 h value set as 1. ** indicates P<0.01 and * indicates P<0.05.

A number of genes have been shown to be required for autophagy in Arabidopsis and several are upregulated under conditions that induce autophagy [50]. Previously, we have shown that under starvation, oxidative, salt and osmotic stresses, autophagy induction is correlated with the upregulation of the *AtATG18a* gene (At3g62770) [5], [6]. In the RNAi-*AtTOR* plants, autophagy was observed even under control conditions. Therefore the expression of the *AtATG18a* gene was analyzed in these plants. *AtATG9* (At2g31260) and the *AtATG8* gene family are also essential for autophagy in *Arabidopsis* [8], [9]; the expression of *AtATG9* and several *AtATG8* [*AtATG8b* (At4g04620), *AtATG8e* (At2g45170), *AtATG8f* (At4g16520), *AtATG8h* (At3g06420)] genes was also analyzed. WT and RNAi-2 seeds were germinated on control MS plates and grown for one week, RNA was extracted from 8 mm of the root tips where autophagy was seen and RT-PCR was performed. As shown in Figure 3A, in the RNAi-2 plants, where the *AtTOR* gene was partially silenced, the *AtATG18a* and *AtATG9* transcript levels were increased compared with WT plants. In contrast, no increases were observed for the *AtATG8b*, *AtATG8e*, *AtATG8f* and *AtATG8e* genes. 18S RNA was used as a control for equal RNA levels in each sample. The expression level of each gene was quantified by densitometry of bands from at least three independent RT-PCR experiments, with the expression in WT plants set to 1. The mRNA level of both *AtATG18a* and *AtATG9* was significantly higher in the RNAi-2 plants than in WT, but no difference was seen in expression of any of the *AtATG8* genes tested compared with WT plants (Figure 3B).The same RT-PCR experiment was also performed with the RNAi-3 line, with identical results to the RNAi-2 line (data not shown).

Previously, *AtATG18apro: GUS* transgenic plants were generated, in which the GUS reporter gene was expressed under the control of the *AtATG18a* promoter as an alternative way to observe the expression of *AtATG18a* [19]. In these plants, GUS activity is very low under control conditions, but increases substantially upon induction of autophagy. To confirm the RT-PCR result, *AtATG18apro: GUS* plants were crossed with RNAi-2 plants. The seeds were germinated on MS plates and grown for one week; the seedlings were then collected and submerged in GUS staining solution for 16 h, destained in 70% (v/v) ethanol for 16 h and observed with a light microscope (Figure 3C). In the *AtATG18apro: GUS* plants, the roots have no visible GUS activity under control conditions. In contrast, in the RNAi*-*2×*AtATG18apro: GUS* plants, GUS activity is evident throughout the roots. This indicates that the *AtATG18a* gene is up regulated in the RNAi*-*2×*AtATG18apro: GUS* plants.

To further quantify the GUS staining results in Figure 3C, GUS activity was measured using the fluorometric substrate 4-methylumbelliferyl-β-D-glucuronide (MUG) [51]. MUG can be hydrolyzed by GUS to produce the fluorochrome 4-methyl umbelliferone (MU). *AtATG18apro: GUS* and RNAi*-*2×*AtATG18apro: GUS* seeds were germinated and grown on MS medium for 7 days with the plates oriented perpendicular to the ground. 0.05 g of root tissue was excised, followed by measurement of GUS activity by monitoring the fluorescence of MU at 0 h, 0.5 h, 1 h, 2 h, 4 h, 6 h, and 16 h (Figure 3D). In both *AtATG18apro: GUS* and RNAi*-*2×*AtATG18apro: GUS* plants, the fluorescence increased gradually over time. However, the fluorescence increase was much greater in the RNAi*-*2×*AtATG18apro: GUS* plants. After 6 h, the fluorescence in RNAi*-*2×*AtATG18apro: GUS* plants was more than 3 fold higher than in the *AtATG18apro: GUS* plants, and increased to almost 8 fold higher after 16 h. This again shows that the RNAi*-*2×*AtATG18apro: GUS* plants have higher GUS activity than the *AtATG18apro: GUS* plants, which is consistent with the GUS staining results.

These results together indicate that the *AtATG9* and *AtATG18a* genes are up-regulated in the RNAi-*AtTOR* plants in the absence of stress.

### AtTOR-regulated autophagy is dependent on *AtATG18a*

**Figure 4:**
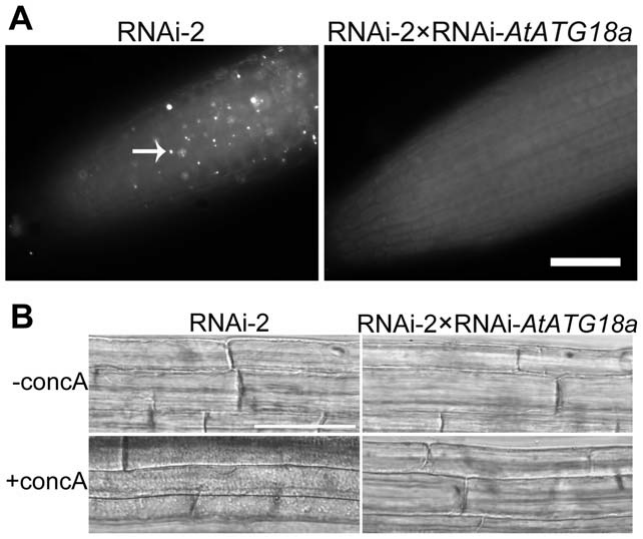
*AtTOR*-regulated autophagy is dependent on *AtATG18a*. **A** MDC staining was performed on seven-day-old RNAi-2 and RNAi-2×RNAi-*AtATG18a* seedlings and observed by fluorescence microscopy. The white arrow indicates an MDC-stained autophagosome. **B** Seven-day-old RNAi-2 and RNAi-2×RNAi-*AtATG18a* seedlings were transferred to liquid MS medium containing 1 µM concanamycin A (+concA) or DMSO (-concA) for 12 h, followed by DIC microscopy. Scale bar = 50 µm.

Since AtATG18a is required for autophagosome formation during stress and senescence, RNAi-*AtATG18*a plants with reduced *AtATG18a* transcript level are defective in autophagosome formation [5]. To determine whether AtTOR*-*regulated autophagy requires AtATG18a, the RNAi-2 plants were crossed with RNAi-*AtATG18a* plants. The seeds were germinated on MS plates and grown for one week, followed by MDC staining. As shown in Figure 4A, RNAi-2 plants have constitutive autophagy in control conditions, while RNAi-2×RNAi-*AtATG18a* plants have no observable autophagy. This indicates that autophagy induced upon inactivation of AtTOR requires AtATG18a, in common with stress-induced autophagy.

To further confirm the absence of autophagosome formation in the RNAi-2×RNAi-AtATG18a plants, conc A was used to assess the accumulation of autophagic bodies in the vacuole. Seven-day-old RNAi-2 and RNAi-2×RNAi-AtATG18a seedlings were transferred to medium containing 1 µM conc A or DMSO as a control for 12 h, and then observed by DIC microscopy (Figure 4B). In control conditions, spherical structures were absent in both RNAi-2 and RNAi-2×RNAi-AtATG18a vacuoles. After treated with conc A, spherical structures accumulated in the RNAi-2 vacuoles but not the RNAi-2×RNAi-AtATG18a vacuoles. These data again suggest the constitutive formation of autophagosomes in the RNAi-2 but not the RNAi-2×RNAi-AtATG18a plants. This confirms that AtTOR-regulated autophagy is dependent on AtATG18a.

### NADPH oxidase inhibitor does not inhibit autophagy in RNAi-*AtTOR* plants

Previous results showed that autophagy is induced by nutrient deprivation, senescence, high salinity, oxidative and osmotic stresses [5], [6], [19]. All of these conditions elevate the cellular ROS (reactive oxygen species) levels, which can act as signal molecules to activate stress response and defense pathways [52], [53]. Plasma membrane NADPH-dependent oxidase is a major source of signaling ROS. The NADPH oxidase inhibitors DPI (diphenylene iodinium) and imidazole [54] inhibit nutrient and salt stress-induced autophagy, whereas autophagy induced by osmotic stress is insensitive to these inhibitors, indicating that autophagy is regulated by NADPH oxidase-dependent or –independent pathways, determined by the induction conditions [19].

**Figure 5:**
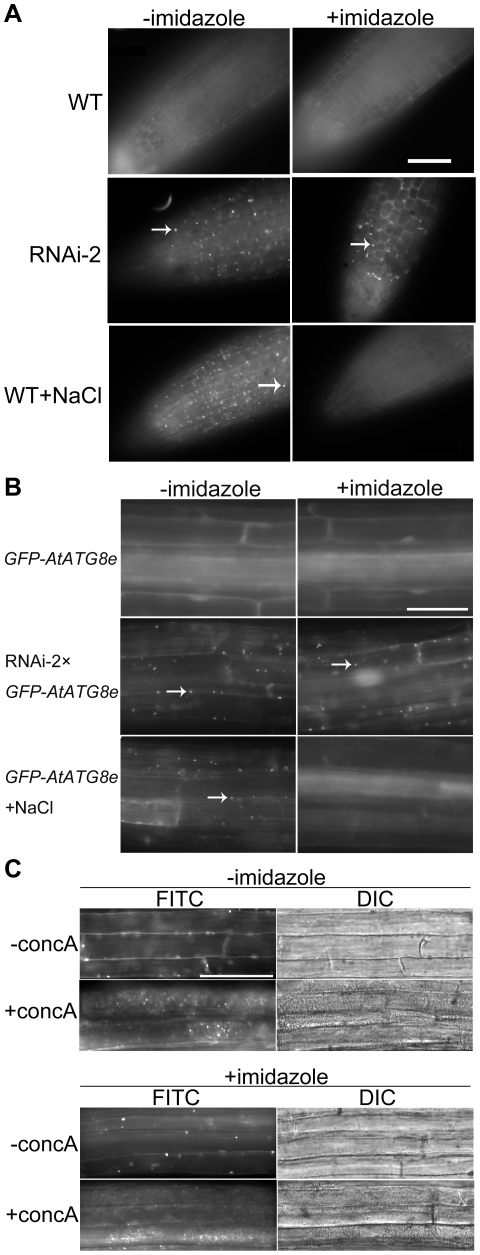
NADPH oxidase inhibitor does not inhibit autophagy in RNAi-*AtTOR* plants. **A** 7-day-old WT and RNAi-2 seedlings were transferred to liquid MS medium plus or minus 20 mM imidazole for 4 h, or WT seedlings were transferred to liquid MS medium plus 0.16 M NaCl plus or minus 20 mM imidazole for 4 h. Autophagosomes were detected by MDC staining. Arrows indicated MDC-stained autophagosomes. **B** 7-day-old *GFP-AtATG8e* and RNAi-2× *GFP-AtATG8e* seedlings were transferred to liquid MS medium plus or minus 20 mM imidazole for 4 h, or *GFP-AtATG8e* seedlings were transferred to liquid MS medium plus 0.16 M NaCl plus or minus 20 mM imidazole for 4 h. Autophagosomes were detected by fluorescence microscopy via a FITC-specific filter. Arrows indicated GFP-labeled autophagosomes. Scale bar = 50 µm. **C** Seven-day-old RNAi-2× *GFP-AtATG8e* seedlings were transferred to liquid MS medium containing 1 µM concanamycin A (+concA) or DMSO (-concA) as a solvent control for 12 h, or liquid MS medium plus 20 mM imidazole containing 1 µM conc A or DMSO for 12 h followed by both fluorescence microscopy and DIC microscopy. Scale bar = 50 µm.

To determine whether *AtTOR* works downstream or upstream of NADPH oxidase in the autophagy signaling pathway, the effect of the NADPH oxidase inhibitor imidazole [54] on RNAi-*AtTOR* plants was tested. One week old RNAi-2 and WT seedlings, germinated on control MS plates, were transferred to liquid MS medium plus or minus 20 mM imidazole. WT seedlings transferred to liquid MS medium plus 0.16 M NaCl plus or minus 20 mM imidazole were used as positive controls. To observe autophagosomes, MDC staining was performed (Figure 5A). For the WT seedlings, no autophagosomes were observed either with or without imidazole treatment under the control conditions, demonstrating that as expected, imidazole does not induce autophagy. Upon exposure to 0.16 M NaCl, numerous autophagosomes were seen in WT plants in the absence of imidazole, whereas in the presence of imidazole, no autophagosomes were present, confirming that imidazole inhibits autophagy induction under salt stress conditions, consistent with previous results [19]. For the RNAi-2 plants, imidazole had no effect on the constitutive autophagy seen in these lines, with numerous autophagosomes seen both in the presence and the absence of this inhibitor. This indicates that the NADPH oxidase inhibitor imidazole does not inhibit the observed autophagy in the RNAi-2 plants.

To confirm the MDC staining results (Figure 5A), one week old RNAi-2× *GFP-AtATG8e* and control *GFP-AtATG8e* seedlings were transferred to liquid MS medium plus or minus 20 mM imidazole. *GFP-AtATG8e* seedlings transferred to liquid MS medium plus 0.16 M NaCl plus or minus 20 mM imidazole were used as positive controls. Autophagosomes can be directly visualized via GFP fluorescence (Figure 5B). In the control condition, no autophagosomes were observed either with or without imidazole treatment. Autophagy was induced in the presence of 0.16 M NaCl, whereas adding imidazole inhibited the autophagy induction. In RNAi-2× *GFP-AtATG8e* seedlings, GFP-labeled autophagosomes were seen both with and without the imidazole. These results are consistent with the MDC staining, again suggesting that imidazole does not inhibit autophagy in the RNAi-2 plants.

To confirm that the GFP-AtATG8e structures in the RNAi-2 plants are autophagosomes, one week old RNAi-2× *GFP-AtATG8e* seedlings were incubated in the presence of 1 µM conc A or DMSO for 12 h, each plus or minus 20 mM imidazole. Autophagic bodies were then observed by both fluorescence microscopy using a FITC filter and differential interference contrast (DIC) microscopy (Figure 5C). In both the presence and absence of imidazole, many punctate GFP-AtATG8e signals were observed. After adding conc A, in both the presence and absence of imidazole, many punctate GFP-labeled spherical structures were observed. By comparing the GFP fluorescence with DIC images, both GFP-AtATG8e and the spherical structures in DIC images were localized inside the vacuoles. This indicates that conc A treatment led to the accumulation of autophagic bodies in the vacuoles both with and without imidazole treatment, again confirming that the NADPH oxidase inhibitor imidazole does not inhibit the constitutive generation of autophagosomes in the RNAi-2× *GFP-AtATG8e* plants. Together, these results suggest that *AtTOR* is either downstream of NADPH oxidase, or works in an NADPH oxidase-independent pathway.

## Discussion

Although several autophagy-related genes have been identified and characterized in plants during the past decade, the upstream regulatory components in the autophagy pathway are still unclear. Autophagy has been reported to be activated in the unicellular green alga *Chlamydomonas reinhardtii* upon rapamycin treatment [42], suggesting that TOR negatively regulates autophagy in this species. However, due to the insensitivity of *Arabidopsis* to rapamycin and the lethality of the knockout mutant, the study of *TOR* function in the autophagy pathway in land plants has been difficult [36], [39]. To overcome these obstacles, we generated RNAi-*AtTOR* transgenic plants to reduce *AtTOR* transcript level. These lines were first confirmed to have reduced growth phenotypes (Figure 1B), consistent with previous research [40], and the reduced root growth phenotype was enhanced upon nitrogen starvation. TOR regulates multiple pathways to coordinate the response to nutrient availability, and decreased TOR activity during starvation is predicted to both increase the activity of the autophagy pathway and decrease growth-promoting pathways such as translation [55]. In two independent RNAi-*AtTOR* lines, constitutive autophagy was observed in roots under control conditions, in contrast to WT plants in which autophagosomes are rarely seen in the absence of stress (Figure 2). The autophagy-related genes *AtATG18a* and *AtATG9* were shown to have an increased transcript level in the RNAi-*AtTOR* lines (Figure 3A, 3B). *AtTOR*-regulated autophagy was dependent on *AtATG18a*, as RNAi-*AtTOR*×RNAi-*AtATG18a* plants do not show constitutive autophagy (Figure 4). Finally, an NADPH oxidase inhibitor did not inhibit autophagy in RNAi-*AtTOR* plants as it does under nutrient and salt stress conditions (Figure 5), suggesting that *AtTOR* is either downstream of or in a pathway that is parallel to NADPH oxidase.

There is considerable evidence that TOR is an upstream regulator of autophagy in numerous species. Tor suppresses starvation-induced autophagy in the *Drosophila* fat body [56] and yeast Tor controls autophagy by responding to nutrient availability [32]. In the green alga *Chlamydomonas reinhardtii*, which is sensitive to rapamycin, inhibition of TOR by rapamycin leads to an increase in vacuole size and formation of ATG8-decorated autophagosomes, consistent with a role in regulation of autophagy[42], [57]. In plants, TOR was shown previously to sense nutrient availability and regulate plant growth [40]. However, little evidence has directly shown a relationship between TOR and autophagy in multicellular plants. In this study, we provide evidence that TOR also controls autophagy, in addition to growth, in plants.

When autophagy is induced by stress conditions, autophagosomes are observed in the roots [5], [6]. Autophagosomes were observed in the area close to the root tip in the RNAi-*AtTOR* seedlings, primarily in the division and elongation zones. TOR is most highly expressed in rapidly dividing and growing tissues such as meristems, embryos and endosperm [36]. It is therefore likely that the decreased expression of the *AtTOR* gene has the greatest effect on rapidly growing and expanding cells and therefore caused the induction of autophagy in these cells. It is clear from this and previous research [36], [40] that TOR signaling is only partially suppressed in the RNAi lines described, and a complete disruption of TOR activity may be necessary to see the full effect of TOR inhibition on autophagy. In addition, the fluorescence assays used are technically difficult in shoot tissues due to the higher autofluorescence, making assessment of possible induction of autophagy in shoot cells problematic. It is possible that autophagy is also activated in rapidly growing shoot tissues in the RNAi lines. The GUS staining results shown in Figure 3C suggests an increased expression of *AtATG18a* throughout most of the root in the RNAi-*AtTOR* plants; this suggests that *AtTOR* activity is affected in upper parts of the root as well as root tips. One explanation is that the increased expression of *AtATG18a* in certain root regions does not necessarily correspond to the formation of autophagosomes. Further reduction of *AtTOR* expression level may be required for autophagosome formation, or other factors may also contribute to regulation of autophagy induction.

In general, Tor proteins function as Ser/Thr protein kinases [22]. Tor signals through a combination of direct phosphorylation of downstream targets and repression of phosphatase activity [58]. Some possible substrates have already been identified in plants, such as a meiosis signaling molecule AML1 (Arabidopsis Mei2-like 1) [59] and EBP1 (ErbB-3 epidermal growth factor receptor binding protein) [60] in *Arabidopsis*, and a translation regulator S6K (ribosomal p70 S6 kinase) [39], [61], but their relationship to autophagy induction is not known. The Atg1/Atg13 complex has been shown to be a Tor substrate in yeast and metazoans. Tor functions by phosphorylating Atg13 in a nutrient-dependent manner, although the regulation and functions of Tor, Atg1 and Atg13 are divergent when comparing different species, complicating the generation of an overall model for TOR function [32], [33], [34], [35]. In *Arabidopsis*, three putative Atg1 homologues and two putative Atg13 homologues have been identified [62], but their functions and interactions with TOR are still unknown. Our study shows that at least some *AtATG* genes are under the control of *AtTOR* in the autophagy pathway. However, to better understand and further investigate the components in the autophagy pathway, future experiments may focus on testing autophagy induction by inactivating or activating *AtTOR* and identifying its downstream targets.

## Materials and Methods

### Plant materials and growth conditions

Arabidopsis seeds were surface sterilized with 0.1% (v/v) Triton X-100 and 33% (v/v) bleach solution for 20 min, followed by cold treatment for at least 2 days. Seedlings were grown at 22°C under long day conditions (16 h light) on nutrient solid MS medium [Murashige–Skoog Vitamin and Salt mixture (Caisson, North Logan, UT, USA), 1% (w/v) sucrose, 2.4 mM MES (pH 5.7) and 0.8% (w/v) phytagar].

### Generation of RNAi-*AtTOR* transgenic plants

The RNAi-*AtTOR* construct was generated as described by Chuang and Meyerowitz [43]. Sense and antisense fragments of *AtTOR* were amplified by RT-PCR (reverse transcription-polymerase chain reaction) using gene-specific primers (Table 1). The sense fragment, a 1-kb *GUS* spacer gene fragment, and the antisense fragment were ligated into the plant T-DNA binary vector pCGN and driven by the Cauliflower Mosaic Virus 35S promoter. The RNAi construct was introduced into *Agrobacterium tumefaciens* strain GV2260 by electroporation [63], and then into *Arabidopsis thaliana* Columbia-0 plants by Agrobacterium-mediated transformation using the floral dip method [44]. The expression level of the *AtTOR* gene in each transformant was determined by RT-PCR and homozygous T2 transformant seeds with reduced *AtTOR* mRNA level were used for further studies.

**Table 1:** Primers used for generating the RNAi-*AtTOR* construct and for RT-PCR analysis of *AtTOR*, *AtATG18a*, *AtATG9* and *AtATG8*s.

	Forward	Reverse
Sense	EcoRV5′-AGCGGATATCATGTCTACCTCGTCGCAATC-3′	XbaI5′-TCGCTCTAGACCAATCTCCGTCAACTCATC-3′
Anti-sense	EcoRI5′-AGCAGAATTCATGTCTACCTCGTCGCAATC-3′	BamHI5′- ACGCGAATCCCCAATCTCCGTCAACTCATC-3′
*AtTOR*	5′-TCAGTCAGGCGAAATCTACTCTACT-3′	5′-TATCCTAGCAATGATTTGAGGTAGC-3′
*AtATG18a*	5′-TCGCGTCGACTCCTTCAAATCATTCTTCCATG-3′	5′-TCGCTCTAGATTAGAAAACTGAAGGCGGTTT-3′
*AtATG9AtATG8bAtATG8eAtATG8fAtATG8h*	5′-GTCGACATGAGCAGTGGGCATAAGGGTCCAAATG-3′ 5′-AGATCTATGGAGAAGAACTCCTTCAAGC-3′5′-AGATCTATGAATAAAGGAAGCATCTTT- 3′5′-AGATCTATGGCAAAAAGCTCGTTCAAG-3′ 5′-AGATCTATGAAATCGTTCAAGGAACAATACAC-3′	5′-GGGCCCTCACCGTAATGTGGTGCTTGATGTTG-3′ 5′-TCTAGATTAGCAGTAGAAAGATCCACCAAATGT-3′ 5′-TCTAGATTAGATTGAAGAAGCAACGAA-3′ 5′-TCTAGAAGCAAGAGGTCTCTATTATGGAGATCC-3′ 5′-TCTAGATCAACCAAAGGTTTTCTCACTGCT-3′

RNAi-2×RNAi-*AtATG18a* plants were generated by crossing RNAi-2 plants with RNAi-*AtATG18a* plants [5]. RNAi-2×*AtATG18apro: GUS* plants were generated by crossing RNAi-2 plants with *AtATG18apro: GUS* plants [19].

### RT-PCR analysis of *AtTOR*, *AtATG18a* and *AtATG9*

Total RNA was extracted using the TRIzol reagent (Invitrogen, Carlsbad, CA, USA) and followed by DNase I treatment. The final RNA concentration was determined using a NanoDrop ND-1000 spectrophotometer; 1 µg RNA was used to generate cDNAs using Superscript III reverse transcriptase (Invitrogen, Carlsbad, CA, USA). An oligo dT primer was used for *AtATG18a* and the *AtATG8*s (*b*, *e*, *f*, *h*), gene specific primers were used for *AtTOR* and *AtATG9* (Table 1). Gene specific primers used for PCR are also shown in Table 1. The PCRs were run for 28 cycles with annealing temperatures of 50, 55, 55 and 60°C and extension times of 1, 1, 1 and 4 min for *AtTOR*, *AtATG18a*, *AtATG8*s and *ATG9*, respectively.

RT-PCR signals were quantified by densitometry. Individual bands were analyzed using Quantity One software (Bio-Rad Laboratories; Hercules, CA) using the volume analysis function. The relative signals were calculated with the wild-type control value set as 1 for each gene individually. The results shown are an average of at least 3 independent experiments.

### MDC staining and microscopy

Wild-type and RNAi-*AtTOR* seedlings were stained with MDC as previously described [47]. Seedlings were incubated with 0.05 mM MDC for 10 mins, washed 3 times with phosphate buffered saline (PBS) and observed using a Zeiss Axioplan II compound microscope equipped with Axio Cam HRC digital imaging system (Carl Zeiss Inc., Göttingen, Germany). MDC fluorescence was visualized using a DAPI-specific filter and GFP fluorescence was visualized using a FITC-specific filter.

### Concanamycin A Treatment

Seven-day-old seedlings grown on MS plates were transferred to MS liquid medium containing 1 µM concanamycin A or dimethyl sulfoxide (DMSO) as a solvent control for 12 to 16 h in the dark. The roots were mounted in water and then observed by fluorescence and differential interference contrast (DIC) microscopy.

### GUS staining

Seedlings were collected and submerged for 16 h in the following staining solution: Triton/ethanol stock (Triton X-100: ethanol: water; 1∶4∶5), 0.5 M KPO_4_ buffer (pH 7.0), 0.1 M ferricyanide solution (pH 7.0), 0.1 M ferrocyanide solution (pH 7.0), 10 mg/ml bromo-4-chloro-3-indolyl-ß-D-glucopyranoside in dimethyl sulphoxide (5∶470∶2∶2∶25). Plants were washed once with 70% (v/v) ethanol, destained in 70% ethanol for 16 h and observed by light microscopy [64].

### Fluorometric assay

Fluorometric reactions for analysis of GUS activity were performed according to Jefferson et al. [51]. 0.05 g of root tissue was homogenized in 50 µl extraction buffer (0.1 M Tris-HCl pH 7.5, 0.3 M sucrose, 1 mM EDTA, 0.1 mM PMSF) with liquid nitrogen. Plant extracts were centrifuged for 5 min at 4°C at 14,000 rpm. The supernatants were added to 1 mM MUG solution and at regular time intervals the reactions were terminated with 0.2 M Na_2_ CO_3_ solution. Fluorescence was then measured with a BIO-TEK Synergy HT multi-detection microplate reader, with excitation at 360 nm and emission at 460 nm. The microplate reader was calibrated with freshly made 4-methyl umbelliferone (MU) standards of 0.5 nm, 5 nm, 25 nm and 50 nm.

### Inhibitor treatment

7-day-old seedlings grown on nutrient solid MS medium were transferred to MS liquid medium plus or minus 20 mM imidazole for 4 h.
